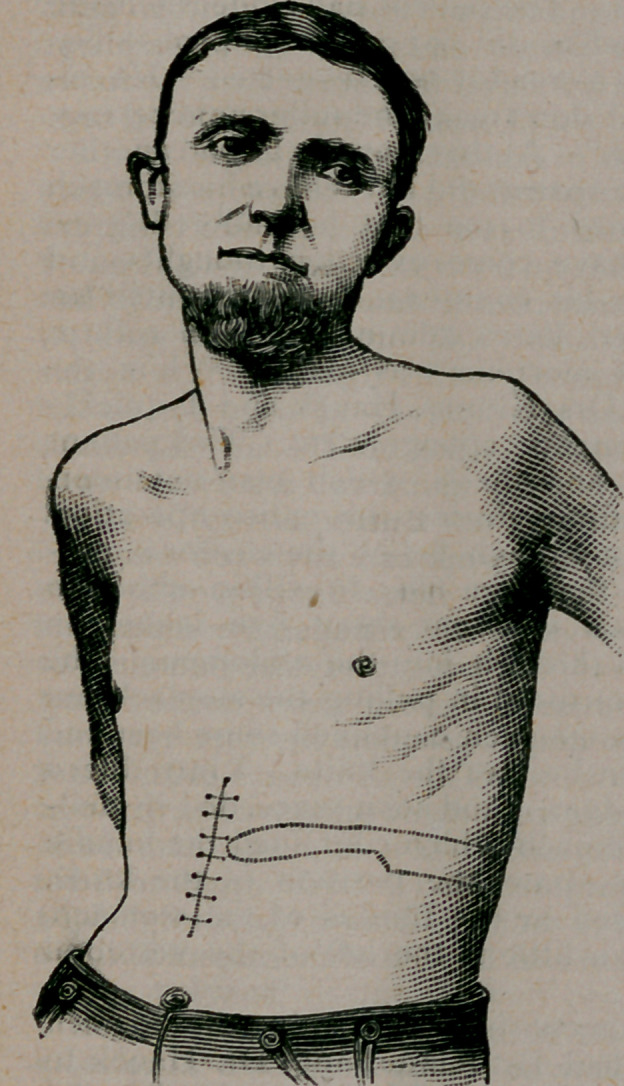# Gastrotomy for the Removal of a Swallowed Knife

**Published:** 1887-02

**Authors:** Augustus C. Bernays

**Affiliations:** Louisville, Ky.; Member German Society Surgeons of Berlin; Professor of Anatomy St. Louis College Physicians and Surgeons


### GASTROTOMY FOR THE REMOVAL OF A SWALLOWED KNIFE. RECOVERY OF THE PATIENT. WITH ILLUSTRATION.*

BY AUGUSTUS C. BERNAYS,

A. M., M. D., Heidelberg; M. R. S., England; F. R. M. S.. London; Member German Society Surgeons of Berlin; Professor of Anatomy St. Louis College Physicians and Surgeons.

In compliance with your request I submit the following report of a most remarkable case for the benefit of your many thousands of subscribers.

Joseph Hoffmann, a German tailor, aged thirty-eight, was amusing his wife and children with various tricks and funny performances, at his home No. 1207 S. Broadway, on the evening of November 17, 1886. They were sitting around a table and, being somewhat exhilerated, Hoffmann intended to close his entertainment by his *chef d' œuvre*, of sword swallowing, in which performance he is an expert. He had frequently pushed pokers, canes and handles of ladles down his gullet before, but on this evening he chose an ordinary case-knife. He intended to make the knife disappear in his throat and then pull it out with his fingers, after the spectators had sufficiently admired his skill and daring. The first act of this programme succeeded admirably, the artist pushed the knife down into his œsophagus, handle foremost, his chin being raised and head thrown back, so that the canal into which the knife was pushed formed a straight line. Suddenly, while in this position, the knife escaped the control of the performer. Amid the agonizing screams of the family and of the victim, the knife was carried down into the stomach by the contractions of the pharyngeal and œsophageal muscles. It was swallowed exactly in the same way as any other substance which is introduced into the fauces. The screams in Hoffmann's dwelling attracted the neighbors and the policeman on the beat. The latter telegraphed for an ambulance intending to remove Hoffmann to the City Hospital, whilst others summoned medical aid. The first medical man to arrive was the family physician, Dr. Hugo Kinner, one of the busiest practitioners of the south-side. After he had assured himself of the condition of his patient he quieted him and stepping to the nearest telephone sent for me. Dr. Kinner and I were soon in earnest consultation by means of the electric current, and it was settled that I should drive down to Hoffman's residence, see him at once and come prepared to operate. The well known occulist, Dr. Chas. Barck, and Dr. Eugene Hauck, accompanied me to the scene of the accident.

When we arrived at the house, Hoffmann was having a violent spell of vomiting, and presented the appearance of a person frightened almost out of his wits. The patient had evidently made up his mind that he must die and he did not grasp the probability of being saved by an operation as readily as I expected. He refused, saying: "Oh, let me die! don't make me suffer unnecessary pain, you can't help me anyhow." At that moment he had another severe spell of vomiting, but the spasms did not relieve his stomach of any its contents, and it seemed to me that he suffered great pain. A change seemed to have come over his thoughts and with an expression of hope on his countenance, he mounted an improvised operating table. Dr. Barck administered chloroform and a hypodermic injection of morphia was made. While the patient was being narcotized, Drs. Kinner, Hauck and I quickly prepared the necessary instruments, sponges, etc. The patient passed into a remarkably quiet anæsthesia, which was not interrupted by a single spell of vomiting during the entire operation. I began the first incision about an inch below the ensiform process and cut straight down on the linea alba to within about an inch from the umbilicus. This was cut about five inches in length and was quickly carried through into the abdomen. The second step of the operation consisted in pulling the stomach out of the abdominal incision. The stomach contained some beer and the remnants of a light supper, besides the knife. I introduced my whole left hand into the abdomen and soon succeeded in pulling out the pyloric end of the stomach which contained the handle of the knife. The dotted line shows the position of the knife. The end of the blade was located in the fundus of the stomach, near the angle of the ninth rib, a little to the left of the vertebral column.

The third step of the operation consisted in opening the stomach and extracting the knife. I had Dr. Kinner and Dr. Hauck to grasp the anterior wall of the stomach with two "army" bullet forceps, about an inch apart on eitheir side of the handle of the table knife, and pull up the stomach so that none of the contents could escape after I had opened it. I then cut through the walls of the stomach upon the handle of the knife within, making a straight cut between the two forceps not exceeding five-eighths of an inch in length. I then pushed the stomach back over the knife handle about half an inch, and grasping it with my fingers, easily extracted it without a drop of the gastric contents escaping. Thus far the operation had consumed scarcely five minutes.

The most difficult and tedious part of the operation was the suture of the small cut in the stomach. The success of the operation, my patient's life, depended upon this procedure, and I performed it with the utmost care after the following method: I first united the edges of the cut by five interrupted sutures ; four of these sutures embraced the peritoneal and muscular layer. I allowed only the middle one to pass through the mucous membrane of the stomach. They were less than one-eighth of an inch apart, and were made with the finest kind of cat-gut. The ends of the sutures were cut close. I next introduced eight ordinary Lembert sutures over and between the five sutures. These, when tied, completely buried out of sight the direct sutures. These latter were made with the thinnest kind of twisted Chinese silk, and their ends were also cut off cl se. It will be seen that the sutures which were employed by me are very similar to the ones used by Billroth, of Vienna, in his operations on the stomach.

I now replaced the stomach in the abdomen. There was little or no bleeding, and the toilet of the abdominal cavity was very simple. The operation was finished by sewing up the external wound in the usual way. I applied about eighteen silk sutures and dressed the wound in the same manner that am I accustomed to, after ovariotomy. The dressings were held in place by an elastic web bandage. The patient was carried to his bed. He rallied quickly after having been under the influence of chloroform about an hour. The knife had been in his stomach less than an hour, before the operation.

The after-treatment was conducted by Dr. Kinner in a most judicious but strict manner, and was followed by a most brilliant result. The patient never vomited at all after the operation ; his temperature reached 100° F. only on one occasion, for a short time, and his pulse never exceeded 86°. He was given a spoonful of water about every two or three hours during the first four days, but large nutrient enemata of peptonized milk, beef tea, etc. were given three times a day. The entire wound healed by first intention. I removed the stitches on the fifth day. The patient *got up on the tenth day* and was discharged from medical attendance on the fourteenth day. The photograph, from which this plate is copied, was taken on December 6, nineteen days after the operation. The patient is as well in every respect as he was previous to the accident.

The distinguishing feature of this case is: Firstly, the prompt manner in which the operation was performed, the knife having remained in the stomach only about one hour. Secondly, the knife which I removed seems to have been the longest object, which has been successfully removed from the stomach by gastrotomy—nine and a half inches. Thirdly, there are some minor pecularities in regard to the method of suture and the employment of antiseptics, which differ from former cases.—*Medical Brief.*

---

" My pa," said one small boy, is a preacher, and is sure to go to heaven." " Huh! " said the other small boy, " that ain't nothin'. My pa is a doctor, and can kill your old pa."

## Figures and Tables

**Figure f1:**